# A Common Trinucleotide Repeat Expansion within the Transcription Factor 4 (*TCF4*, E2-2) Gene Predicts Fuchs Corneal Dystrophy

**DOI:** 10.1371/journal.pone.0049083

**Published:** 2012-11-21

**Authors:** Eric D. Wieben, Ross A. Aleff, Nirubol Tosakulwong, Malinda L. Butz, W. Edward Highsmith, Albert O. Edwards, Keith H. Baratz

**Affiliations:** 1 Department of Biochemistry and Molecular Biology, Mayo Clinic, Rochester, Minnesota, United States of America; 2 Department of Biomedical Statistics and Informatics, Mayo Clinic, Rochester, Minnesota, United States of America; 3 Department of Laboratory Medicine and Pathology, Mayo Clinic, Rochester, Minnesota, United States of America; 4 Institute for Molecular Biology, University of Oregon and Oregon Retina, Eugene, Oregon, United States of America; 5 Department of Ophthalmology, Mayo Clinic, Rochester, Minnesota, United States of America; University of Florida, United States of America

## Abstract

Fuchs endothelial corneal dystrophy (FECD) is a common, familial disease of the corneal endothelium and is the leading indication for corneal transplantation. Variation in the transcription factor 4 (*TCF4*) gene has been identified as a major contributor to the disease. We tested for an association between an intronic TGC trinucleotide repeat in TCF4 and FECD by determining repeat length in 66 affected participants with severe FECD and 63 participants with normal corneas in a 3-stage discovery/replication/validation study. PCR primers flanking the TGC repeat were used to amplify leukocyte-derived genomic DNA. Repeat length was determined by direct sequencing, short tandem repeat (STR) assay and Southern blotting. Genomic Southern blots were used to evaluate samples for which only a single allele was identified by STR analysis. Compiling data for 3 arms of the study, a TGC repeat length >50 was present in 79% of FECD cases and in 3% of normal controls cases (p<0.001). Among cases, 52 of 66 (79%) subjects had >50 TGC repeats, 13 (20%) had <40 repeats and 1 (2%) had an intermediate repeat length. In comparison, only 2 of 63 (3%) unaffected control subjects had >50 repeats, 60 (95%) had <40 repeats and 1 (2%) had an intermediate repeat length. The repeat length was greater than 1000 in 4 FECD cases. The sensitivity and specificity of >50 TGC repeats identifying FECD in this patient cohort was 79% and 96%, respectively Expanded TGC repeat was more specific for FECD cases than the previously identified, highly associated, single nucleotide polymorphism, rs613872 (specificity = 79%). The TGC trinucleotide repeat expansion in TCF4 is strongly associated with FECD, and a repeat length >50 is highly specific for the disease This association suggests that trinucleotide expansion may play a pathogenic role in the majority of FECD cases and is a predictor of disease risk.

## Introduction

Fuchs corneal dystrophy (FECD) is a common, progressive, late onset disease affecting the endothelial cell monolayer on the internal surface of the cornea. The phenotypic hallmark of the disease is corneal guttae, which are collagenous excrescences of the endothelial basement membrane [1]. The disease course is marked by an increasing density of guttae and attrition of the endothelial cells. Loss of the fluid-pumping function of the endothelium cells results in thickening and hydration of the corneal stroma, a decrease in corneal transparency and a resultant loss of vision. The condition is uncommonly diagnosed prior to the fourth decade of life, but about 5% of United States (US) adults over 40 years old exhibit guttae. [2] Corneal edema due to severe FECD develops in a small proportion of patients with guttae but is the most common indication for corneal transplantation in the US, accounting for almost 12,500 of the approximately 46,000 grafts performed in 2011 and contributing to an unknown proportion of the more than 14,400 grafts done annually for corneal edema after other eye surgery or for failed previous corneal grafts [3].

The inheritance pattern of FECD has long been considered an autosomal dominant trait with variable expressivity. [4], [5], Other than smoking and a low body mass index, there is little evidence that environmental or modifiable risk factors play a role in the natural history of the disease. [7] Mild disease is asymptomatic, whereas visual loss from severe disease is treatable only by corneal transplantation.

Mutations in several genes, including *LOXHD1* [8] and *SLC4A11* [9], [10] *and TCF8* [11] have been associated with a small proportion of FECD cases, and variation in the *COL8A2* gene is responsible for a rare early-onset Fuchs-like disease. [12] The genetic basis of the majority of the cases remains unexplained. Baratz and colleagues described results of a genome-wide association study (GWAS) that revealed a highly significant association between single nucleotide polymorphisms (SNPs), most notably rs613872, in the transcription factor 4 (*TCF4*) gene and typical, late onset FECD. [13] The TCF4 gene (also called *ITF2* and *SEF-2*) codes for the helix-loop-helix transcription factor E2-2. The TCF4 gene on chromosome 18 should not be confused with the t-cell factor 4 (*TCF7L2*) gene on chromosome 10, which is also called *TCF4* by some authors and in some databases.

Breschel et al [14] (1997) previously described an unstable trinucleotide repeat within the third intron of TCF4. They noted expanded, unstable alleles in approximately 3% of the population that they studied. We have investigated the association of FECD with this trinucleotide repeat expansion in a series of 66 FECD cases and 63 matched control subjects. Our data suggest that 52 of these cases (79%) have expanded alleles. Only 2 of 63 control subjects (3%) show evidence of an expansion at this locus. Our work suggests the likelihood that FECD is a trinucleotide repeat expansion disorder in the majority of cases.

## Results

**Figure 1:**
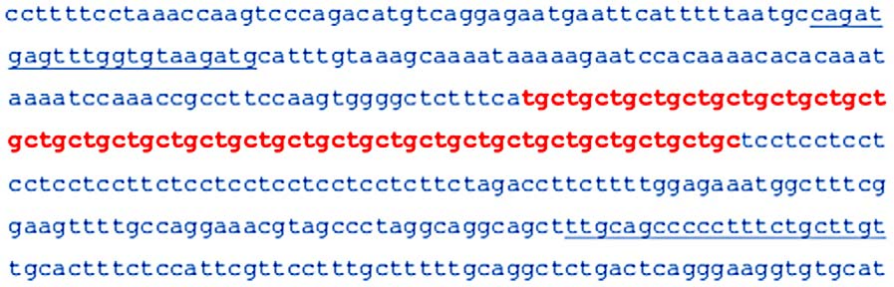
DNA sequence surrounding the trinucleotide repeat in the intron of the *TCF4* gene on chromosome 18. The trinucleotide repeat region is shown in red, and PCR primer sequences used for sizing the repeat region are underlined. This version of the sequence comes from the human reference sequence and contains 25 TGC repeats.

Partial sequence of the third intron of the *TCF4* gene from GenBank is shown in Figure 1. This version of the sequence contains 25 tandem complete copies of a TGC repeat (shown in red), lying between a TCA trinucleotide on the 5′ extremity and a series of TCC repeats on the 3′ end.

### Discovery set

In the discovery set of experiments, PCR primers flanking the TGC repeat were used to amplify samples of genomic DNA from 10 cases and 10 control subjects. Sizing of the PCR products by agarose gel electrophoresis revealed longer PCR products in each of the FECD patient samples than were found in any of the control samples.

**Figure 2:**
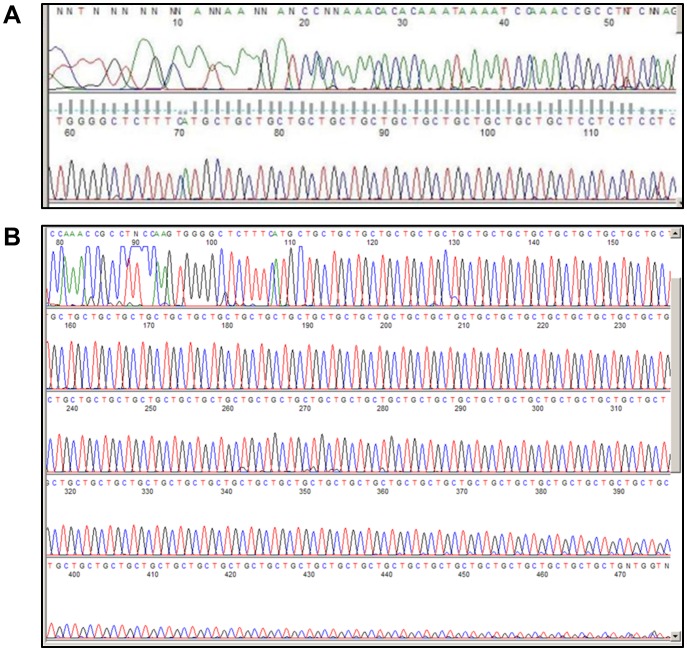
Sanger DNA sequencing of DNA samples of a normal control (A) and a FECD patient with two expanded alleles (B).

Direct DNA sequencing of the PCR products confirmed that the increased size of the products produced from the DNA of patients with FECD was due to expansions of the TGC trinucleotide repeat. The sequence of a control sample containing two 12 repeat alleles is shown in Figure 2A. The sequence of the products from a FECD case with two expanded alleles confirms the presence of amplified alleles with at least 85 repeats of the TGC sequence in the shortest allele (Figure 2B).

**Figure 3:**
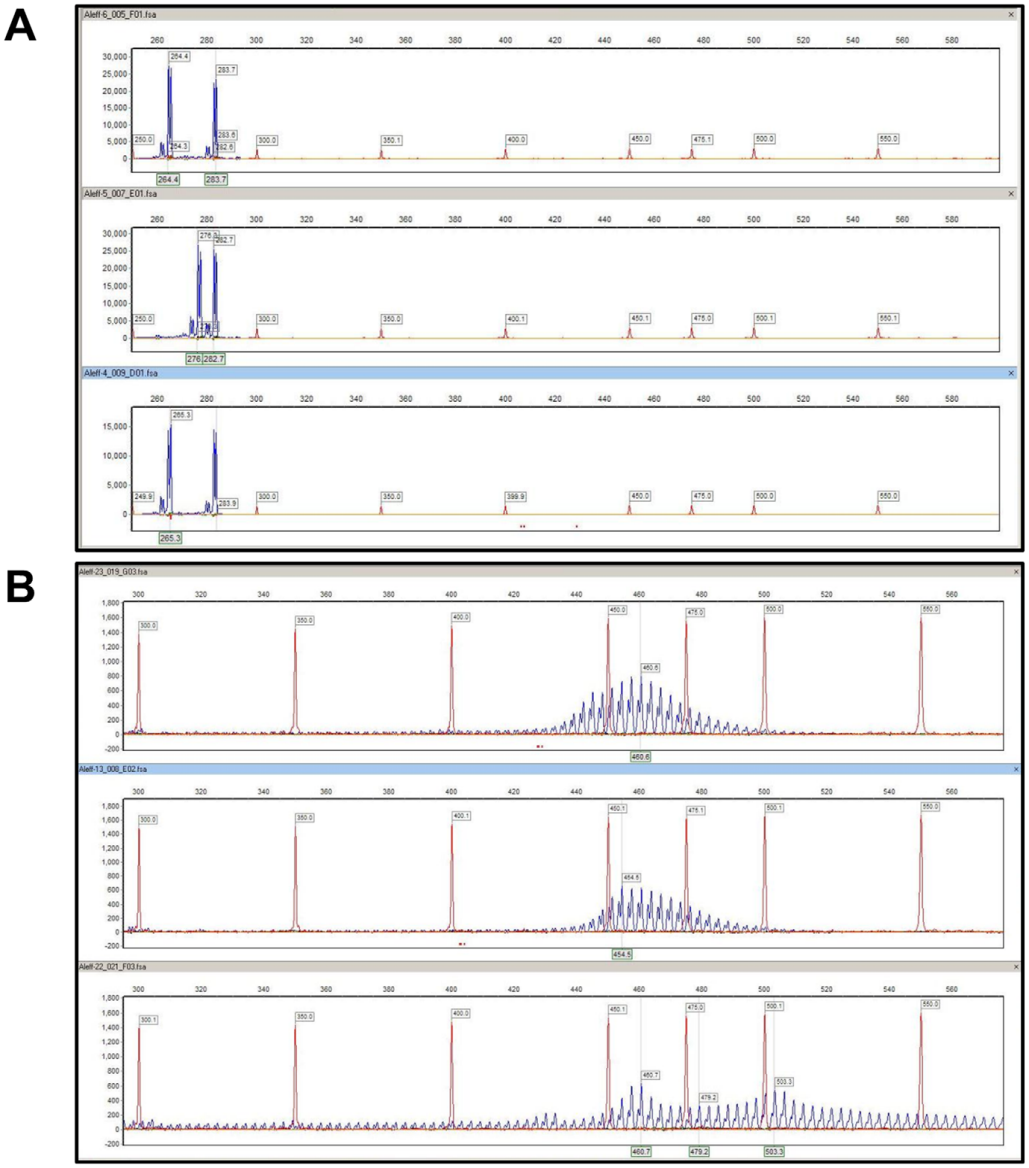
STR analysis of PCR amplicons of 3 normal control (A) and 3 FECD patients (B). The bottom panel is the analysis of a FECD patient with 2 expanded alleles.

To increase the sensitivity and accuracy of the sizing of the products from this region, we used capillary electrophoresis to size fluorescent PCR products containing the expanded repeat (STR assay). These results (Figure 3 and Table 1) confirmed the initial conclusion that all ten of the FECD patients in the discovery set had expanded trinucleotide repeats. The mean size of the longest allele measured by STR in the discovery set was 88 repeats. The longest repeat size observed in the ten control subjects from this sample set contained only 31 repeats of the TGC sequence. In contrast, the FECD patients had repeat sizes of up to 100 repeats, and every patient in this group had at least one allele with more than 50 repeats.

**Table 1:** A comparison of TGC repeat length in TCF4 between FECD cases and normal controls.

	Size of Longest Trinucleotide Repeat Allele (number of repeats)					
	FECD Cases			Controls		
	>50 repeats*	40–50 repeats	<40 repeats	>50 repeats*	40–50 repeats	<40 repeats
Discovery set	10	0	0	0	0	10
Replication set	20	0	5	1	1	20
Validation set	22	1	8	1	0	30
Total	52	1	13	2	1	60
%	79%	2%	20%	3%	2%	95%

* p<0.001 for FECD cases vs. controls by Fisher's exact test.

### Replication set

To confirm these initial results, an additional 25 FECD participants and 22 participants were analyzed by STR, sequencing, and Southern blotting. Twenty of the 25 FECD samples contained expanded repeats (see Table 1). Significantly, one of the 22 control samples also contained a TGC repeat greater than 50 repeats. One additional control sample had 49 repeats. The mean size of the largest allele in the other 20 control samples was 19 repeats (range 12–34).

### Validation set

A third set of samples from 31 FECD cases and 31 control subjects age- (+/−3 years) and gender-matched was also assayed by STR and Sanger sequencing. Twenty-two of the cases in this group were correctly identified as cases with TGC expansion >50 repeats; and one additional sample from an unaffected control subject was found to have a significant expansion (83 copies in the largest allele). The next largest allele found in this group of controls contained only 36 repeats. This group also included 1 FECD case with 48 repeats by STR analysis. The next largest allele found in this validation group of control samples contained only 36 repeats. Eight samples from FECD cases in this group did not yield evidence of expanded repeats in lymphocyte DNA.

**Figure 4:**
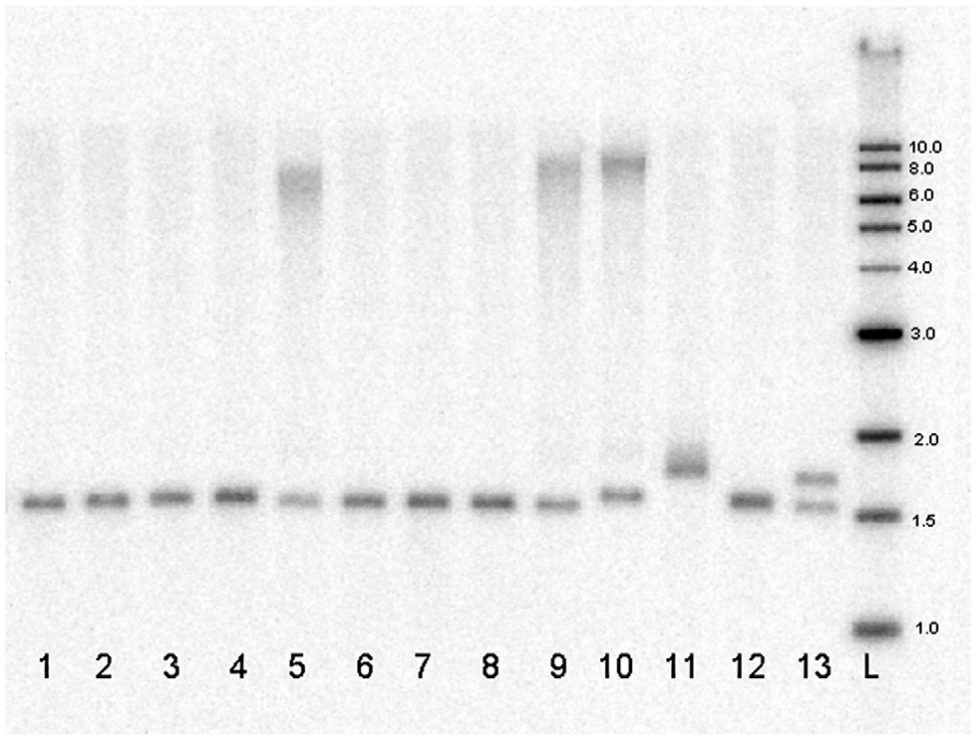
Genomic Southern Blot of DNA samples from normal control (lanes 1, 4, 7 and 8) and FECD patients (lanes 2, 3, 5, 6, 9–11). Lanes 12 and 13 are laboratory control samples that have not been evaluated for FECD. Note that the samples in lanes 2, 3 and 6 are from FECD patients that do not have the repeat expansion. The samples in lanes 5, 9, and 10 are samples from FECD cases with repeat expansion over 1500 repeats. Lane L contains sizing standards.

Genomic Southern blotting was used to evaluate samples from all 3 sample sets showing evidence of only a single allele by STR analysis (Figure 4). Four of these samples from the validation set (lanes 2, 3, 5 and 6) were cases and three (lanes 1, 4, and 7) were controls. From these seven samples, only the sample in lane 5 yielded evidence of expanded repeats by Southern blotting. As shown in Figure 4, this sample yielded a smeared band about 7–8 kb in size, compared to the 1.5 kb bands found in controls. This size is consistent with repeat expansions ranging from 1800 to more than 2000 copies of the repeat. Similarly sized expanded alleles were detected in two other cases (lanes 9–10) from the discovery and replication cohorts, respectively. The sample in lane 11yielded a single band consistent with the 78 repeats measured by STR analysis, indicating 2 copies of similarly-sized, expanded alleles.

**Figure 5:**
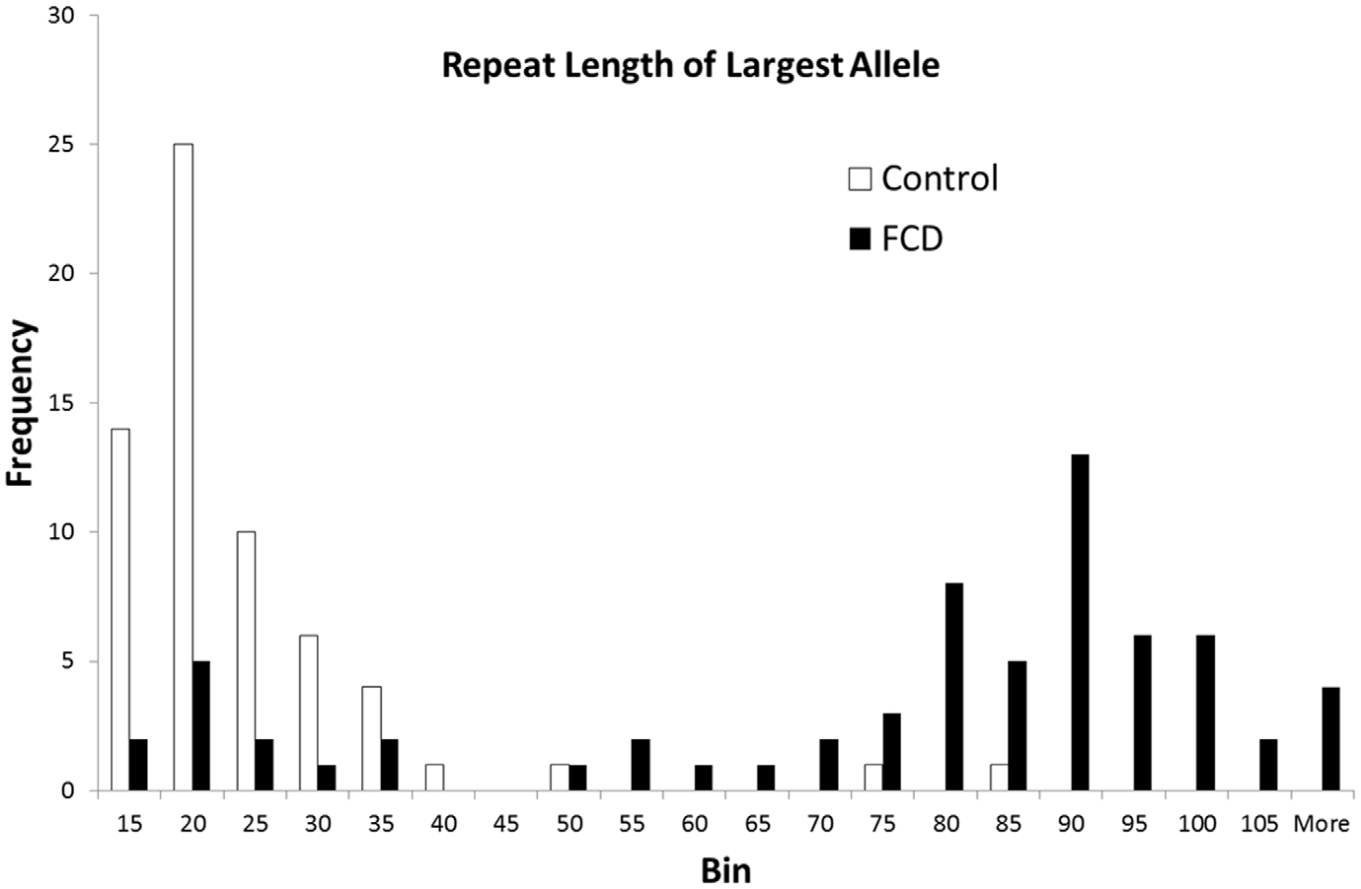
Frequency histogram of the TGC repeat length of the longest allele in all 129 samples. The length of the longest repeat in each sample is shown for both FECD patients (black bars) and normal control subjects (open bars). Note that 3 FECD patients had very long repeat expansions (more than 1500 repeats), as shown in Figure 4.

Figure 5 and Table 1 summarize the repeat length for all samples. More than 50 TGC repeats were present in 54 participants. An expansion of this size was present in 52 of 66 samples from FECD cases, yielding a test sensitivity of 79% in this cohort. Only 2 of 63 (3%) control samples had a similar expansion, so the specificity of >50 repeats identifying an FECD case (52 cases of 54 samples) was 96%. Among 73 samples with <40 repeats, 60 (82%) were from control subjects. In comparison, the sensitivity of at least one copy of the minor rs613872 allele to identify FECD was 83% (55 of 66 FECD cases), but the minor allele was less specific for FECD (55 of 70 samples, specificity = 79%) than a repeat expansion. Among 58 samples with no minor allele, 47 (81%) were control cases (one control subject did not have genotyping for this SNP).

## Discussion

Our data demonstrate a strong association between expansion of a non-coding trinucleotide repeat in the *TCF4* gene and FECD. Unstable trinucleotide expansions cause a small number of neurodegenerative and neuromuscular disorders, including Huntington disease (HD) and variants of myotonic dystrophy (MD), fragile X, Friedreich ataxia, and spinocerebellar ataxia. (Reviewed in Ref # [15], [16])

FECD is a late onset condition that affects as much as 5% of the U.S. population over 40. [2] Studies of *ex-vivo* or cultured human corneal endothelium and mouse models of disease have reported evidence of apoptosis, [17], [18] endoplasmic reticulum stress, [19], [20] and oxidative stress. [21] However, some studies have focused upon cases or models of disease associated with minor genetic variants due to several known mutations unrelated to *TCF4*. Studies of oxidative stress in FECD have described a down-regulation of genes and proteins involved in antioxidant pathways, including nuclear factor erythroid 2-related factor 2 and multiple peroxiredoxins. [21], [22]

Baratz et al., [13] recently demonstrated a very strong relationship between FECD and SNPs in the *TCF4* gene, including the most highly associated SNP rs613872. This finding has been replicated by other groups, [23], [24], [25], [26] and variants in other genes within this region of the genome have been found in small numbers of patients with FECD. [8] Our data indicate that the expansion of the TGC repeat within the *TCF*4 gene is found in a very high proportion of patients with FECD—over 79% of the 66 samples tested. Six of the 52 (12%) FECD patients with expanded repeats had two expanded alleles. We were unable to discern a difference in the disease phenotype or age of onset in these homozygous subjects, possibly due to the small number of cases. However, we are not surprised by the 12% prevalence of homozygous expansion in our study subjects who were selected for severe disease phenotype. Importantly, the expanded repeat was only found in 2 of our control samples. In both cases, a single expanded allele was observed. Notably, 32 of the FECD samples had repeat expansions that were longer than the 83 repeat allele, which was the longest allele noted in controls. Overall, 52 of 54 (96%) subjects that had a repeat length of more than 50 repeats had a clinical diagnosis of FECD and all 3 of the subjects with very large expansions (more than 1500 repeats) were FECD patients. These observations are consistent with the possibility that expansion of a single allele at this location in the genome is a major contributor to the disease process. It is not clear from our data whether the occurrence of expanded alleles in unaffected individuals reflects reduced penetrance, a delayed disease onset or linkage disequilibrium between another causative allele and the expanded repeat. However, given that the gene product of the *TCF4* gene is a transcription factor, it would not be surprising if variants in genes regulated by TCF4 could alter the effect of this expansion on the function of the corneal endothelium.

In the context of the *TCF4* gene, the actual repeat sequence appears to be TGC in all samples we have studied by sequencing. However, we realize that biologically the sequence of this repeat is identical to both the CAG repeats found in Huntington's Disease [27] and the CTG repeats described in Myotonic Dystrophy. [28] One important biological difference between this repeat expansion and those seen in HD and MD is its location within the gene. This expansion is not included in the exons of any of the 47 different transcript variants described in Ensembl. For most of the longer transcripts, the TGC repeat lies in an intron, and for the shorter TCF4 transcripts the repeat lies within the 5′flanking sequence. Hence the expansion will not directly alter the structure of the protein product as it does in HD, and the RNA toxicity hypothesis implicated in MD also seems less likely.

In this regard, it is probably significant that haploinsufficiency of TCF4 expression is thought to be causative for Pitt-Hopkins syndrome, a severe form of neurodevelopmental delay. [29], [30] Thus, if the repeat expansion is causative for FECD, we hypothesize that the effect is to alter the expression of the gene in some way rather than to simply inactivate the gene as in Pitt-Hopkins syndrome. Given the variety of transcripts produced by this gene in a tissue specific manner, it is reasonable to consider the possibility that the repeat expansion alters either the transcription start site or level of expression of specific *TCF4* isoforms that could be required for the maintenance of the corneal endothelium. Other pathogenic effects, including RNA-mediated events, remain possible. The described susceptibility of FECD endothelial cells to oxidative stress might also be consistent with TGC expansion, because the guanine-rich repeats may be particularly susceptible to oxidative damage. Considering the frequency of FECD, the ease of assessing the phenotype, and the availability of diseased tissue from corneal transplant patients, further investigation of this expansion will yield information that will be useful not only to the understanding of this common disease but may also be generalizable to other rare but devastating trinucleotide repeat disorders.

## Methods

This study was approved by the Mayo Clinic Institutional Review Board. Participants were enrolled after signed, informed consent, and the study was conducted in accordance to the Declaration of Helsinki.

### Study participants

A total of 66 FECD participants and 63 control participants were enrolled in the study. There was a preponderance of women in both the affected and control groups (77% female vs. 70% female, p = 0.33), and the FECD group was younger [71 yrs., range = 56–88; vs. 75 yrs, range = 60–91 yrs.; p = 0.01). All participants were Caucasian.

FECD severity was determined by slit lamp examination by one of the authors (KHB or AOE) by using a modified Krachmer scale [31] based upon the more severely affected eye (0 = no guttae; 1 = up to 12 central guttae; 2 = >12 non-confluent central guttae; 3 = 1–2 mm. confluent guttae; 4 = 2–5 mm. confluent guttae; 5 = >5 mm. confluent guttae; 6 = guttae with corneal edema). Subjects selected as cases had at least grade 2 FECD, and all control subjects had no guttae (grade 0). Among the FECD cases, the mean FECD severity was grade 5.5 (range = 2–6), indicating the tendency to select cases with a more severe phenotype for this study. The study was conducted in accordance with the Declaration of Helsinki.

### Genomic DNA

DNA was purified from leukocytes using AutoGen FlexiGene by Qiagen (Valencia, CA.). DNA was resuspended in 1× TE at a final concentration of 250 ng/ul

### Primers

Primers sets are as follows:

5-TCF-Fuchs CAGATGAGTTTGGTGTAAGATG

3-TCF-Fuchs 1 ACAAGCAGAAAGGGGGCTGCAA

5-FAM-TCF-Fuchs FAM CAGATGAGTTTGGTGTAAGATG

3-TCF-Fuchs 1 ACAAGCAGAAAGGGGGCTGCAA

### PCR amplification

PCR assays were conducted using 5′TCF and 3′TCF oligonucleotides specific for *TCF4*. Amplification was performed using an i-cycler Bio-Rad (Hercules, CA.) using Invitrogen Platinum PCR Super Mix High Fidelity (Carlsbad,CA.).

Reaction volume was 50 ul with 100 ng of Genomic DNA and 10 pmoles of each primer. The PCR program was as follows: Hot Start 95°C, 6 min. 1 cycle, then 95°C 1 min., 62°C 1 min., 68°C 3 min. for 35 cycles, and finally 7 min. at 68°C, followed by 4°C hold.

### DNA sequencing

200 ng of DNA was added to 2 ul of Exo-SAP and incubated at 37°C for 15 minutes followed by heat inactivation at 80°C for 15 minutes. For sequencing, 3 ul of DNA was mixed with 1.6 pmoles of 5′ primer. Sequencing was carried out using ABI 3730XL DNA Analyzer (Foster City,CA.)

### Short tandem repeat (STR) assay

For STR analysis, a 5′FAM primer was used in the PCR reaction.

After PCR, 2 ul of DNA was mixed with 12 ul of diluted Map Marker 1000 Bio Ventures Inc. (Murfreesboro, TN.) Gene Scan was carried out using ABI 3730XL DNA Analyzer (Foster City, CA.).

### Genomic Southern blots

Genomic DNA from each patient was digested with EcoRI. Digested genomic DNA (2.5 µg) was then loaded onto a 1.0% agarose gel for overnight electrophoresis at 40 V. After standard capillary gel transfer to the hybridization membrane, 18 ng of the purified probe was radioactively labeled with α-^32^P-dCTP using the PrimeIt II random-primer labeling kit (Agilent Technologies, Santa Clara, CA). The radioactive probes were added to 20 ml of hybridization solution, at a concentration of 1.5×10^6^ cpm/ml. Membranes were placed in the probe/hybridization solution, and hybridization took place overnight at 45°C. After hybridization, the membranes were washed three times in 2× standard saline citrate, 0.1% sodium dodecyl sulfate at 60°C for 30 minutes and then once in 0.2× standard saline citrate, 0.1% sodium dodecyl sulfate at 60°C for 30 minutes. The radioactive membranes exposed to PhosphorImager (Amersham GE Healthcare, Piscataway, NJ) screens overnight, scanned and results analyzed with the ImageQuant 5.0 software (GE Healthcare).

### SNP genotyping

Genotyping of SNP rs613872 was performed either as part of a previous GWAS done at the Center for Inherited Disease Research (Baltimore, Md) on a 370 K Illumina beadchip panel or by TaqMan assays (Applied Biosystems, Foster, CA).
